# Clinical Efficacy of Adipose-Derived Stem Cell-Enriched Lipotransfer Versus Conventional Fat Grafting: A Systematic Review and Meta-Analysis

**DOI:** 10.7759/cureus.105479

**Published:** 2026-03-19

**Authors:** Swastik Sutar, Dhruvesh Patel, Zaid M Al Ani, Purva Mehta

**Affiliations:** 1 Trauma and Orthopaedics, Peterborough City Hospital, Peterborough, GBR; 2 Plastic Surgery, Aberdeen Royal Infirmary, Aberdeen, GBR; 3 Emergency Medicine, Pilgrim Hospital, Boston, GBR

**Keywords:** adipose-derived stem cells, autologous fat grafting, cell-assisted lipotransfer, graft retention, meta-analysis, stromal vascular fraction, volumetric analysis

## Abstract

Autologous fat grafting is a standard procedure for soft tissue reconstruction, yet its clinical utility is often compromised by unpredictable resorption rates. Cell-assisted lipotransfer (CAL), which enriches fat grafts with adipose-derived stem cells (ASCs) or stromal vascular fraction (SVF), has been proposed to stabilise retention through improved neovascularisation and paracrine support. This systematic review evaluates the clinical efficacy, safety, and oncological outcomes of CAL compared to conventional lipofilling.

A systematic literature search was conducted across PubMed, Cumulative Index to Nursing and Allied Health Literature (CINAHL), Web of Science, Excerpta Medica database (EMBASE), and Cochrane Library databases following Preferred Reporting Items for Systematic Reviews and Meta-Analyses (PRISMA) 2020 guidelines. Inclusion criteria were randomised controlled trials (RCTs) and cohort studies comparing enriched versus non-enriched fat grafts in humans. The primary outcome was volumetric retention, while secondary outcomes included complications and oncological safety. A random-effects meta-analysis was performed using Stata 15.1 (StataCorp LLC, College Station, TX) and Simple Meta-Analysis software (Learn Meta-Analysis LLC) to calculate standardised mean differences (SMD), and heterogeneity was assessed using Cochran’s Q and the I² statistic.

Fifteen studies meeting the inclusion criteria were analysed. The meta-analysis indicated a statistically significant improvement in graft survival favouring the enriched groups (SMD 2.44; 95% CI 1.00 - 3.88; p = 0.001). However, extreme statistical heterogeneity was observed (I² = 97.19%), suggesting that the magnitude of benefit is highly variable. Subgroup analysis revealed that enrichment effects were most pronounced in facial applications (SMD 2.95) and studies utilising standard aspiration techniques (SMD 2.94). Conversely, studies employing water-assisted lipotransfer (WAL) showed no significant added benefit to enrichment (SMD -0.22), supporting a harvest-quality ceiling hypothesis where a high-quality baseline harvest may render enrichment redundant. A secondary meta-analysis of patient satisfaction scores revealed no significant difference between the groups (p = 0.52). However, the enriched cohort demonstrated significantly higher costs and longer operative times. Long-term follow-up in post-mastectomy breast reconstruction patients did not demonstrate an increased risk of locoregional recurrence of malignancy.

Current evidence suggests that CAL has the potential to improve volumetric retention compared to conventional fat grafting, particularly in facial reconstruction and when standard aspiration methods are used. However, the efficacy is context-dependent, and the high heterogeneity suggests that CAL cannot be considered uniformly superior across all harvesting techniques and recipient sites. Furthermore, although available evidence does not show an increased oncological risk during the reported follow-up periods, longer-term surveillance remains necessary to confirm these safety profiles. To establish reliable clinical guidelines, future trials must prioritise larger patient cohorts and standardised cell-dosing protocols to minimise procedural variability.

## Introduction and background

The pursuit of the ideal soft tissue filler remains a central theme in plastic surgery. Autologous fat transfer, or lipofilling, has emerged as the preferred choice because it is minimally invasive, biocompatible, and provides a natural contour that synthetic implants fail to replicate [1-3]. However, despite these advantages, the procedure has a major downside. Graft resorption rates have been reported as high as 20% to 80% within the first year of surgery, thus rendering the final aesthetic outcome highly unpredictable [4-11].

This inconsistency represents the primary barrier to the procedure's success. A fat graft is a free tissue transfer that, upon injection, faces an immediate state of oxidative and ischaemic stress [2]. Its survival relies entirely on plasmatic imbibition until recipient neovascularisation is established. Without this rapid vascular support, a sizeable portion of the graft is reabsorbed or undergoes fat necrosis, often leaving patients with underwhelming outcomes and the need for multiple corrective surgeries [2].

To address these limitations, the field has pivoted towards cell-assisted lipotransfer (CAL). This technique augments the standard fat graft with a concentrated dose of adipose-derived stem cells (ASCs). These cells function as a biological chaperone, secreting angiogenic growth factors that accelerate vessel ingrowth and improve the overall take of the graft [2].

For the purposes of this review, the term 'CAL' serves as an umbrella concept encompassing both culture-expanded ASC grafts and stromal vascular fraction (SVF)-enriched grafts. SVF is a heterogeneous mixture of cells comprising vascular endothelial cells, immune cells, and stem cells, obtained via enzymatic or mechanical processing at the point of care. In contrast, ASC enrichment typically involves a homogeneous population of multipotent cells isolated from the SVF via ex vivo culture expansion [5]. While these modalities share a biological rationale rooted in regenerative signalling, they differ significantly in regulatory classification, processing time, and cellular composition. Where data permit, this review distinguishes between these subtypes to evaluate their specific clinical utility.

Current evidence suggests that this enriched fat graft yields superior volume retention and tissue quality, particularly in hostile recipient beds such as radiated breast tissue or severe facial atrophy [2]. This has the potential to reduce the need for secondary surgeries and thus could significantly lower patient morbidity [3]. However, this technique is not without variables. Outcomes are heavily dependent on donor cell viability, recipient site compliance and precision of volumetric assessment tools like three-dimensional computed tomography (3D-CT) or magnetic resonance imaging (MRI) [3,4]. This systematic review evaluates the latest clinical evidence to determine whether ASC enrichment offers a reliable biological edge in modern practice.

Science behind ASCs

ASCs were first described by Zuk et al. as a multipotent, self-renewing cell population isolated from the SVF of lipoaspirate [1]. SVF is a heterogeneous mixture of cells obtained by the mechanical or enzymatic removal of mature adipocytes from the sample. It typically contains approximately 10% of ASCs, situated within a complex niche of vascular endothelial cells, macrophages, smooth muscle cells, lymphocytes, pericytes and fibroblasts [5]. This synergistic microenvironment is critical, as it provides the necessary paracrine signalling to regulate ASCs' function and optimise their regenerative potential under physiological conditions [5].

Harvested via minimally invasive liposuction, these cells demonstrate remarkable biological plasticity. Under specific induction factors, ASCs exhibit multilineage potential, with the capacity to differentiate into osteogenic, adipogenic, myogenic, chondrogenic, and neurogenic tissues [1]. However, in the context of fat grafting, their primary clinical value lies in their potent pro-angiogenic profile. ASCs and their associated endothelial progenitor cells secrete vital growth factors such as vascular endothelial growth factor, fibroblast growth factor, platelet-derived growth factor, and insulin-like growth factor 1, which actively promote neovascularisation and stabilise long-term graft retention [6-8].

The beneficial effect of ASCs on graft survival is primarily attributed to this improved early vascularisation, achieved either through direct ASC differentiation into endothelial cells or via their immunomodulatory and trophic effects [7]. They possess an inherent resistance to hypoxia that allows them to withstand the initial ischaemic shock of the grafting procedure [6,7]. They orchestrate local and systemic biological responses through cell-to-cell communication and paracrine signalling, thereby regulating immune responses, inhibiting apoptosis, and remodelling the extracellular matrix [5]. These properties make them particularly advantageous for regenerating ischaemic tissues where the native vascular bed is compromised [7,9].

Isolating and harvesting ASCs

Lower abdomen and inner thighs are the preferred donor sites due to the ease of surgical access, low cosmetic sensitivity, and a superior concentration of ASCs [6]. In younger patients, abdominal fat has been shown to exhibit lower apoptosis rates and higher proliferative capacity [5,6]. Furthermore, ASCs harvested from these subcutaneous depots are more predisposed to the adipogenic lineage, making them ideal for soft tissue augmentation [5].

Aspiration of adipose tissue inevitably results in a lower stromal cell yield compared to excision, due to mechanical trauma [2]. Consequently, gentle low-pressure liposuction remains the gold standard for obtaining high-quality lipoaspirate. For smaller augmentations under 100 ml, manual aspiration using a 10 ml Luer-Lok syringe is a reliable method [5]. However, for large-scale procedures like breast reconstruction, low-pressure techniques such as the Coleman method or water-assisted lipotransfer (WAL) are preferred to minimise shear stress on the adipocytes [5,8,9].

Once harvested, the lipoaspirate must be processed to concentrate the regenerative population. Because the ASC concentration in aspirated fat is half that of excised tissue, often four times the target graft volume is required to double the physiological stem cell count [7]. Therefore, active enrichment is important to increase the stem cell yield by 3-30% [7]. This is achieved through enzyme-based digestion or mechanical centrifugation, either via manual methods or automated platforms like Celution [9].

Phenotypic characterisation of ASCs

Autologous fat transfer serves as a scaffold upon which more concentrated ASC can organise and differentiate, thus promoting the secretion of soluble factors that enhance angiogenesis, decrease apoptosis, and modulate immune response. To confirm their identity, ASCs are defined by a specific surface marker profile. Flow cytometry analysis characteristically reveals a strong positive expression of mesenchymal markers (CD73, CD90, CD105) and adhesion molecules (CD29) [1]. Conversely, they consistently lack haematopoietic and endothelial lineage markers, showing negative staining for CD14, CD20, CD34, CD45, and CD31 [1].

Application of ASCs

Autologous fat transfer has been adopted as a common surgical procedure for soft tissue reconstruction, not just for volumetric augmentation but for its unique capacity to regenerate tissue quality in complex cases. In post-mastectomy breast reconstruction, CAL offers a distinct advantage over synthetic implants by restoring natural warmth and softness without increasing the risk of oncological recurrence [3,7,12]. Similarly, in the face, high-level evidence supports its efficacy for treating complex congenital deformities like Parry-Romberg disease and craniofacial microsomia, where enriched grafts provide superior structural stability compared to conventional lipofilling [10,11,13-15]. Their trophic effect is equally beneficial in scar management and radiation-induced fibrosis, where the pro-angiogenic activity of the cells helps to revitalise ischaemic tissue and soften restrictive scarring [2,16].

Aim of the study

The primary objective of this meta-analysis is to evaluate the clinical efficacy of CAL compared to conventional autologous fat grafting, with a specific focus on volumetric retention rates. Secondary objectives include the assessment of complication rates, patient satisfaction, and oncological safety, particularly in the context of post-mastectomy breast reconstruction. Furthermore, this study aims to scrutinise the sources of heterogeneity in reported outcomes, specifically differentiating between the efficacy of culture-expanded ASCs versus SVF and analysing the impact of different harvesting techniques on graft survival.

## Review

Materials and methods

Search Strategy

A systematic literature search was conducted across PubMed, Cumulative Index to Nursing and Allied Health Literature (CINAHL), Excerpta Medica database (EMBASE), Web of Science, Cochrane Library, and Ovid Medical Literature Analysis and Retrieval System Online (MEDLINE) for studies published between January 2006 and January 2026. The review followed the Preferred Reporting Items for Systematic Reviews and Meta-Analyses (PRISMA) 2020 guidelines. The search strategy was based on the Population, Intervention, Control, and Outcomes (PICO) framework and combined terms related to fat graft transplantation. To ensure a comprehensive search, we utilised a combination of specific keywords. For the population and intervention, we searched for terms including 'adipose-derived stem cells', 'ASCs', 'stromal vascular fraction', 'SVF', 'cell-assisted lipotransfer', and 'CAL'. These keywords were cross-referenced with terms relating to the surgical procedure, such as autologous fat grafting, lipofilling, adipose tissue transplantation, and fat transfer techniques. To capture the relevant clinical endpoints, the aforementioned terms were combined with outcome-specific keywords, namely graft retention, complications, aesthetic outcomes, functional recovery, and oncological safety. Additional search terms captured procedure-specific concepts, such as expanded cell preparations and enzymatic or mechanical SVF isolation.

Eligible study designs included randomised controlled trials (RCTs), prospective and retrospective cohort studies, meta-analyses, and systematic reviews. Reference lists of included studies and relevant reviews were manually screened to ensure comprehensive coverage. Grey literature, including conference abstracts and unpublished manuscripts, was excluded to maintain methodological quality and reproducibility. Additionally, to include more available research for the review, the reference lists of the included articles were also searched.

Study Selection

All citations retrieved from the database search were imported into a reference manager, and duplicates were removed. Two reviewers (SS and DP) independently screened titles, abstracts, and full texts in three stages. Discrepancies at the title or abstract stage were carried forward to full-text review. Disagreement between reviewers was discussed until a consensus was reached. In the case of persistent disagreement, a senior author (ZA) gave a binding verdict.

Studies were included if they examined patients undergoing autologous fat grafting for aesthetic or reconstructive purposes, compared ASC- or SVF-enriched fat grafting with conventional fat grafting, and reported clinically relevant outcomes such as graft volume retention, complications, patient-reported aesthetic or functional outcomes, or oncological events. Only English-language articles published between 2006 and 2026 were considered in this study. No exceptions to the recipient site were considered.

Studies were excluded if they involved animal or in vitro models, did not include a comparator group, focused solely on procedural techniques without reporting clinical outcomes, or investigated interventions unrelated to ASC or SVF enrichment. Abstract-only publications, editorials, case reports, letters, and conference proceedings were excluded because they did not provide sufficient methodological detail or extractable data. Articles reporting outcomes from fat grafting without cell enrichment were also excluded. Lastly, in case of duplicate research, we selected the article with the largest sample size and the latest publication date.

**Figure 1:**
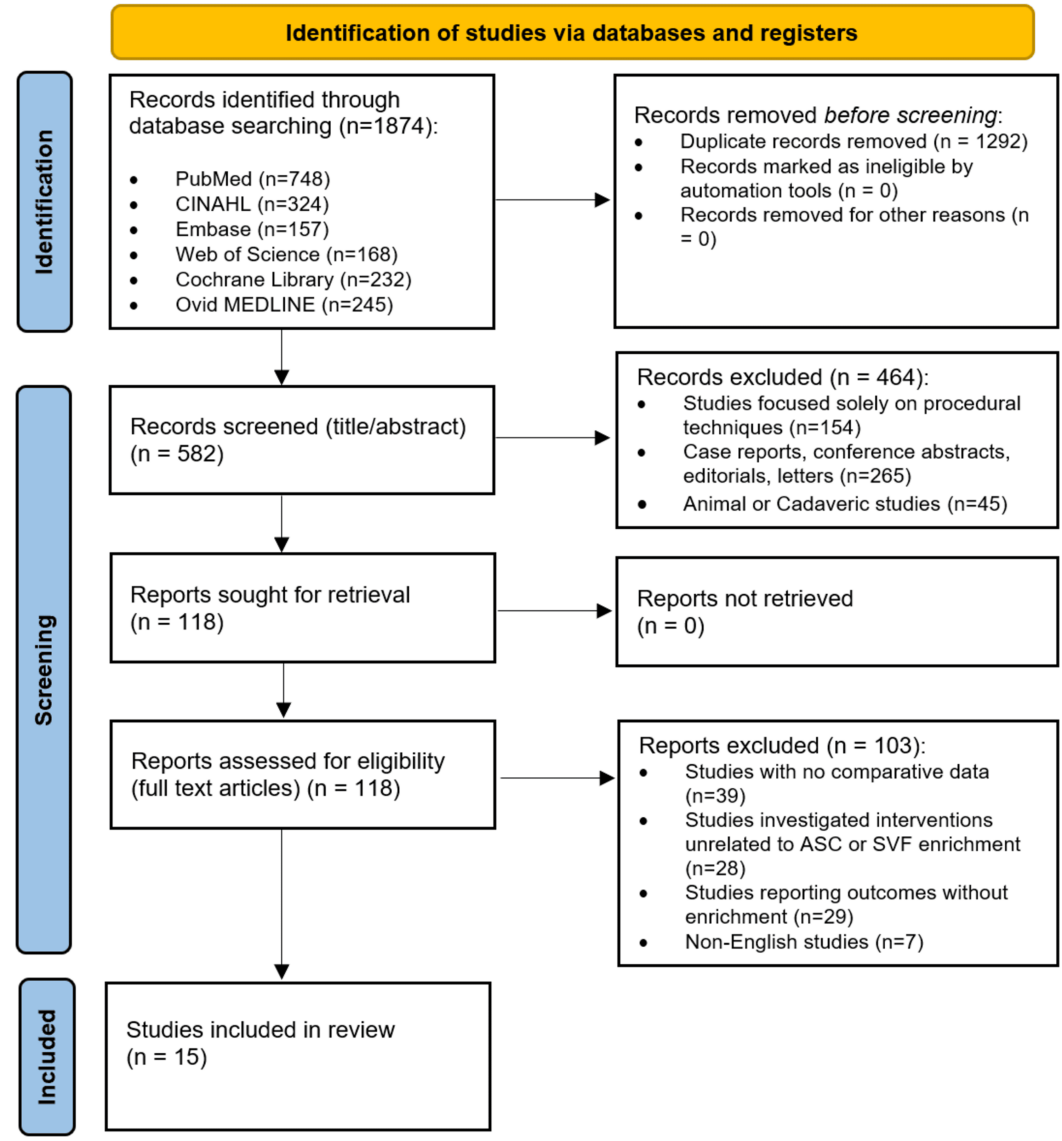
A PRISMA flowchart outlining the study selection process PRISMA: Preferred Reporting Items for Systematic Reviews and Meta-Analyses; CINAHL: Cumulative Index to Nursing and Allied Health Literature; Embase: Excerpta Medica database; MEDLINE: Medical Literature Analysis and Retrieval System Online; ASC: adipose-derived stem cells; SVF: stromal vascular fraction

The full screening process is presented in the PRISMA flow diagram (Figure 1). The initial database search identified a total of 1874 records from PubMed (n = 748), CINAHL (n = 324), EMBASE (n = 157), Web of Science (n = 168), Ovid MEDLINE (n = 245), and the Cochrane Library (n = 232). After removing 1,292 duplicates, 582 records remained for title and abstract screening. During this primary screening phase, 464 records were excluded as they focused solely on procedural techniques (n = 154), were case reports or conference abstracts (n = 265), or were animal or cadaveric studies (n = 45). A total of 118 full-text articles were assessed for eligibility. Of these, 103 were excluded for the following reasons: absence of a control group (n = 39), investigated interventions unrelated to ASC or SVF enrichment (n = 28), reported outcomes without enrichment (n = 29), and non-English language publications (n = 7). Ultimately, 15 studies met the definitive inclusion criteria and were included in the qualitative synthesis [2-4, 7-18].

Data Extraction and Analysis

Data extraction was performed independently by two reviewers (SS and DP) using a predefined Microsoft Excel data sheet (Microsoft Corporation, Redmond, WA, USA). Extracted variables included study design, year of publication, population demographics, intervention and comparator arms, characteristics such as the type of enrichment method used and graft volume, follow-up duration, and the imaging modality used for assessing graft retention. Clinical outcomes were recorded directly from the included studies and encompassed graft volume retention, complication rates, patient-reported outcomes, and oncological events where applicable.

Data accuracy was verified through cross-checking and random spot-checking. Reported statistical findings, including effect estimates and p-values, were taken directly from the original publications. The fat survival rate was our primary endpoint in assessing the efficacy of lipotransfer between the CAL and non-CAL groups. We also analysed complications like necrotic cysts, oil cysts, solid indurations, and malignancy. The temporary adverse reactions, such as redness and swelling, that appeared after surgery were not included in our analysis.

Risk of Bias Assessment

The methodological quality of the included studies was independently assessed by two reviewers using the Effective Public Health Practice Project (EPHPP) tool [19]. This instrument was selected for its versatility in evaluating diverse study designs, including both RCTs and observational cohorts. Studies were evaluated across six domains: selection bias, study design, confounders, blinding, data collection methods, and withdrawals. Based on these parameters, a global rating was assigned. Studies with no weak ratings were classified as strong, those with one weak rating as moderate, and those with two or more weak ratings as weak. While the study protocol was not prospectively registered in the PRISMA database, this rigorous assessment framework was utilised to ensure objective evaluation and to identify potential sources of bias across the evidence base.

Statistical Analysis

We performed a meta-analysis of fifteen studies using a random-effects model, applying the Restricted Maximum Likelihood (REML) estimator, to synthesise continuous outcomes, including fat survival and patient satisfaction rates. Given the anticipated variability in surgical techniques and patient populations, a random-effects model was deemed necessary to account for between-study heterogeneity. The outcomes are expressed as standardised mean differences (SMDs) with their associated 95% confidence intervals (CIs).

Statistical heterogeneity was quantified using the Cochran’s Q statistic and I² index, where I² > 50 was considered indicative of significant heterogeneity. To explore the sources of this variance, subgroup analyses were conducted based on recipient sites (breast vs. face), stem cell enrichment methods (ASCs vs. SVF) and harvesting technique (standard aspiration like Coleman or syringe vs. WAL). Sensitivity analyses were conducted by sequentially omitting individual studies to evaluate the robustness of the pooled effect estimates. Publication bias was statistically confirmed using Egger’s linear regression test. All statistical procedures were performed using Simple Meta-Analysis software (Learn Meta-Analysis LLC) and Stata 15.1 (Stata Corporation, College Station, TX) for advanced bias diagnostics.

Results

Study Characteristics

A total of 15 primary studies were included in this review, enrolling a total of 634 patients (range: six to 206 participants per study). The evidence base comprised a mix of nine RCTs, three prospective cohort studies and three retrospective cohort studies. The characteristics of these studies, published between 2008 and 2023, are summarised in Table 1. The level of evidence across the included literature was high, with nine studies (60%) classified as Level 2 and six studies (40%) classified as Level 3. These trials originated from diverse global centres, including Denmark, Italy, South Korea, Brazil, and China, providing a broad evidence base for this study.

**Table 1:** Demographic and Methodological Characteristics of the Included Studies RCT: randomised controlled trial; SG: stem group (intervention); CG: control group; SD: standard deviation; BMI: body mass index (kg/m^2^); NR: not reported; M/F: male/female ratio.

Study	Year	Country	Study Design	Sample Size	Age, years Mean +/- SD, Range	Gender, M/F	BMI, Kg/m^2^ Mean +/- SD, Range	Level of Evidence
Kolle et al. [7]	2013	Denmark	RCT	10	28.4 +/ 3.2 (SG); 28.4 +/ 3.2 (CG)	2/8	24.7 +/- 0.7 (SG); 24.7 +/- 0.7 (CG)	2
Calabrese et al. [12]	2018	Italy	Prospective cohort study	169	48.8, 34-61 (SG); 50.3, 33-69 (CG1); 47.7, 33-60 (CG2)	0/169	NR	3
Koh et al. [14]	2012	South Korea	RCT	10	28 (SG); 28 (CG)	5/5	NR	2
Kolle et al. [8]	2020	Denmark	RCT	12	29, 21-42 (SG); 32.5, 24-39 (CG)	0/12	19.2, 18-22.5 (SG); 20.8, 19.7-23.8 (CG)	2
Peltoniemi et al. [9]	2013	Finland	Prospective cohort study	18	51, 29-58 (SG); 39, 33-63 (CG)	0/18	23.4, 20.3-32.5 (SG); 23.4, 20.3-25.9 (CG)	3
Tanikawa et al. [10]	2013	Brazil	RCT	14	12.1 +/- 2.2 (SG); 18.7 +/- 7.6 (CG); p=0.06	5/9	<25	2
Yin et al. [11]	2020	China	RCT	50	35.5 +/- 8.2, 22-50 (SG): 35.3 +/- 8.1, 20-51 (CG)	0/50	21.2 +/- 1.8 (SG); 21.6 +/- 1.9 (CG)	2
Glowinski et al. [3]	2022	Denmark	RCT	10	33 +/- 7.19 (SG); 33 +/- 7.19 (CG)	0/10	24.3 +/- 2.07 (SG); 24.3 +/- 2.07 (CG)	2
Tissiani et al. [4]	2016	Brazil	RCT	19	49.6 (SG) 50.4 (CG)	0/19	25.8 (SG) 25.3 (CG)	2
Roshdy et al. [13]	2022	Egypt	RCT	15	49	0/15	>20	2
Li et al. [15]	2013	China	Retrospective cohort study	38	26	0/38	29.5	3
Bashir et al. [16]	2019	Pakistan	RCT	37	30 (SG) 21 (CG)	11/26	NR	2
Chang et al. [17]	2013	China	Retrospective cohort study	20	27.5	8/12	NR	3
Chiu et al. [18]	2019	Taiwan	Retrospective cohort study	206	37	0/206	NR	3
Yoshimura et al. [2]	2008	Japan	Prospective cohort study	6	42.5	2/4	NR	3

The patient population was predominantly female (94.8%, n = 601), with nine studies exhibiting a female sex bias driven by the inclusion of studies focused primarily on breast reconstruction. Baseline demographics were well-balanced between the study arms. The mean age of the participants was 35.2 years (95% CI 29 - 41.32 years) in the intervention group and 34.50 years (95% CI 28.9 - 40.03 years) in the control group, with no statistically significant difference (p = 0.57). Seven studies reported the mean BMI of 24.01 kg/m² (95% CI 20.96 - 27.07 kg/m²) in the stem group and 24.23 kg/m² (95% CI 21.60 - 26.86 kg/m²) in the control group (p = 0.43). 

ASC Enrichment Methodologies

The technical protocols for graft enrichment and adipose processing are summarised in Table 2. Of the included studies, nine (60%) utilised SVF enrichment, while six (40%) employed culture-expanded ASCs. The abdomen was the most frequent donor site (n = 14), followed by the thighs (n = 6) and flanks (n = 1). The mean volume of harvested fat was approximately 160 mL (range: 50 - 311 mL).

**Table 2:** Adipose Stem Cell Enrichment and Processing Methodologies ASC: adipose-derived stem cells; SVF: stromal vascular fraction; GA: general anaesthesia; LA: local anaesthesia; rpm: rotations per minute; g: grams; ml: millilitres; SG: stem group; CG: control group; NR: not reported.

Study	Enrichment (ASC/SVF)	Harvested site and volume	Extraction method, anaesthesia	Centrifugation rate	Sample Processing	Enrichment ratio or concentration	CD marker expression	Recipient site
Kolle et al. [7]	ASC	Abdomen; 50 ml	Coleman technique of liposuction; GA	3000 rpm for 3 minutes	Ex vivo expansion (14 days) using human platelet lysate	8.58 X 10^8^ ASCs/mL	CD90(95%), CD73(95%), CD105 (70-85%), HLA-DR (2-4%)	Posterior upper arm (subcutaneous bolus)
Calabrese et al. [12]	SVF	Abdomen, thigh, flanks, gluteal or inner knees; NR	Coleman technique of liposuction; GA	3000 rpm for 3 minutes	Automated enzymatic digestion (Celution system) using proteolytic enzymes	NR	NR	Breast (mastectomy flaps during 2nd stage reconstruction)
Koh et al. [14]	ASC	Abdomen; 100 g	Coleman technique of liposuction; GA	1500 rpm for 5 minutes	3 hours of enzymatic digestion (type 1 collagenase)	1 X 10^7^ ASCs/mL	CD105, CD73, CD90 positive; CD45, CD34, CD14, CD11b, CD79a, CD19, and HLA-DR negative	Face (hemifacial atrophy secondary to Parry-Romberg disease)
Kolle et al. [8]	ASC	Abdomen, thigh or hip; 100ml	Coleman technique of liposuction; LA	3000 rpm for 3 minutes	Ex vivo expansion (human platelet lysate)	20 X 10^6^ ASCs/mL	CD90, CD73, CD105 positive; CD34, CD45, CD14, CD19, HLA DR negative	Breast (cosmetic augmentation)
Peltoniemi et al. [9]	ASC	Abdomen; 311 (240-365) ml	Water jet-assisted liposuction; LA	Centrifugation not used	Celution 800/CRS system	NR	CD73, CD90, CD105 positive	Breast (cosmetic augmentation)
Tanikawa et al. [10]	SVF	Lower abdomen; NR	Coleman technique of liposuction; NR	700 g for 3 minutes	20-min enzymatic digestion (collagenase type 1A)	1:1 ratio of freshly isolated SVF	CD73, CD90, CD105, CD29 positive; CD45 and CD31 negative	Face (craniofacial microsomia)
Yin et al. [11]	SVF	Inferior abdomen; 200-240 (SG) 50-60 (CG)	Coleman technique of liposuction, GA	NR	Mechanical centrifugation	1-3 X 10^7^/ml ASC	CD29, CD44, CD73, CD90, CD105	Face (rejuvenation)
Glowinski et al. [3]	ASC	Abdomen and Thigh; 200	Water jet-assisted liposuction; LA	100g for 3 minutes	Ex vivo expansion (human platelet lysate)	10 X 10^6^ ASCs/mL	CD73, CD90, CD105	Breast (cosmetic augmentation)
Tissiani et al. [4]	SVF	Abdomen; NR	Coleman technique of liposuction, GA	335g for 2 minutes	30 minutes of enzymatic digestion (collagenase 1A)	2:1 enrichment ratio	CD29 (70.09%), CD31 (20.56%), CD34 (43.92%), CD45 (8.59%), CD73 (40.36%), CD90 (79.01%), CD105 (35.81%)	Breast (secondary reconstruction)
Roshdy et al. [13]	SVF	Outer thigh; NR	Coleman technique of liposuction, GA	3000 rpm for 3 minutes	Enzymatic digestion (collagenase type 1A)	3.6 - 6 X 10^5^	CD105(85.7%), CD44(56.7%), CD73(60.1%), CD90(40.6%), CD34(0.1%), CD45(0.2%)	Temples (augmentation)
Li et al. [15]	SVF	Lower abdomen and thigh; NR	Coleman technique of liposuction; NR	1000 rpm for 3 minutes	Enzymatic digestion (collagenase)	NR	NR	Face (cosmetic augmentation)
Bashir et al. [16]	ASC	Abdomen; NR	Syringe aspiration using a 10-ml Luer-Lok syringe; NR	1000 rpm for 10 minutes	Enzymatic digestion (collagenase)	NR	NR	Face
Chang et al. [17]	SVF	Abdomen; NR	Coleman technique of liposuction; NR	1000 rpm for 3 minutes	Enzymatic digestion (collagenase)	NR	NR	Face
Chiu et al. [18]	SVF	Abdomen, flank, hip, thigh, calf; NR	Coleman technique of liposuction; NR	800g for 5 minutes	Enzymatic digestion (collagenase)	4.07 X 10^7^ ASCs/ml	NR	Breast (augmentation)
Yoshimura et al. [2]	SVF	Abdomen; NR	Suction-assisted liposuction; GA	700g for 3 minutes	Mechanical centrifugation	NR	NR	Face (Parry-Romberg and lupus lipoatrophy)

Harvesting techniques varied significantly between studies. The Coleman technique served as the standard harvesting method for most trials (n = 12), typically paired with centrifugation at 3,000 rpm for three minutes (n = 4) to isolate the adipocytes. In contrast, WAL was used in two studies as the primary method of harvesting [3,9]. By continuously infiltrating tumescent fluid at high pressure while suctioning at low pressure, WAL automatically rinses and filters the graft without damaging the fat cells [9]. This method was noted to be significantly faster (saving 90-150 minutes), cheaper, and theoretically safer due to a reduced risk of contamination [9]. Notably, to maximise cell viability, general anaesthesia (n = 7) was preferred over local infiltration (n = 3) in multiple high-quality studies to mitigate the potential cytotoxicity of lidocaine and improve the graft uptake [9].

The isolation of the stem cell-rich stroma from the lipoaspirate was achieved through three primary methodologies. The Celution system, an automated closed-system device, was utilised in two studies to process the lipoaspirate and isolate the SVF at the point of care [9,12]. Standard enzymatic digestion with collagenase (Type 1A or Type 2) was the most common technique for SVF isolation, employed in nine studies. In contrast, ASC enrichment protocols (n = 6) typically involved ex vivo culture expansion over a period of 14 to 17 days, often utilising human platelet lysate or foetal bovine serum to reach supra-physiological concentrations, ranging from 3.6 × 10^5^ to 8.5 × 10^8^ ASCs/ml.

Cell characterisation was performed in most studies using flow cytometry to assess the stem cell immunophenotype. ASC-enriched grafts demonstrated high expression of mesenchymal markers (CD90, CD73, and CD105), whereas SVF populations were more heterogeneous, frequently expressing haematopoietic and endothelial markers such as CD34 and CD45. Tissiani et al. found a positive correlation between the expression of CD31, CD73, CD90, and CD105 and the volumetric persistence of the graft. However, CD90 was the only marker to show a statistically significant correlation (p = 0.03) with fat survival, suggesting its potential utility as a predictive biomarker for graft take [4].

Recipient sites were primarily the face, the breast, and the digits. The face was the most common recipient site (n = 7), utilised for correcting congenital deformities like Parry-Romberg syndrome or for cosmetic rejuvenation. The breast was the second most frequent site (n = 6), primarily used for post-mastectomy reconstruction.

Follow-Up and Assessment Protocols

The mean follow-up period across the included studies was 16·2 (95% CI 5·5-26·8) months, with a range extending from four to 84 months. While a one-stage approach was performed in the majority of trials (n = 11), Koh et al. employed a two-stage approach for Parry-Romberg syndrome. This involved an initial injection of non-enriched fat to correct base atrophy, followed by the administration of ASC-enriched fat two weeks later to achieve a targeted 30% overcorrection [14].

The primary efficacy endpoint was the fat survival rate, defined as the ratio of residual graft volume to the initial volume of fat injected. Secondary measures comprised cosmetic outcome, patient satisfaction (measured using the Visual Analogue Scale, transplant vessel density, complication rate (categorised as major and minor events), malignancy potential and cost analysis.

Objective volumetric measurement was the standard across high-quality trials, utilising high-precision techniques to quantify graft retention. MRI-based volumetry with axial 3D sequences was considered the gold standard (n = 5), particularly for breast reconstruction, due to its reproducibility and ability to simultaneously assess graft viability and exclude complications such as oil cysts or fat necrosis [4,7]. Other validated methods included ultrasound biomicroscopy (n = 3), 3D laser scanning with Geomagic software (n = 2), and CT-based workspace analysis with Philips Extended Brilliance (n = 4). Koh et al. noted that while CT is effective for volumetric confirmation, it was less ideal than non-ionising 3D camera systems due to the risk of radiation and higher procedural costs [14].

Detailed clinical outcomes and complication profiles are summarised in Table 3.

**Table 3:** Clinical Outcomes, Volumetric Retention, and Complications Following Cell-Assisted Lipotransfer ASC: adipose-derived stem cells; SVF: stromal vascular fraction; WAL: water-jet assisted liposuction; MRI: magnetic resonance imaging; CT: computed tomography; SG: stem group; CG: control group; VISIA: video image skin interpretation and analysis; SD: standard deviation.

Study	Intervention (n)	Comparator (n)	Number of operations	Volume injected, ml Mean +/- SD, Range	Follow up, months	Measurement method	Volume gained, ml Mean +/- SD, Range	Fat survival rate, % Mean, Range	Other outcomes	Complication
Kolle et al. [7]	ASC-enriched fat graft (10, one arm)	Non-enriched fat graft (10, one arm)	2	28.2 +/- 1.13 (SG); 28.6 +/- 1.17 (CG); p=0.78	4	MRI	23 +/- 1.22 (SG); 4.66 +/- 0.75 (CG)	80.9 +/- 6 (SG); 16.3 +/- 7.2 (CG); p<0.0001	No significant difference in vessel density (20.86 vs. 26.32 vessels/mm^2^, p=0.39)	Significantly less fat necrosis in enriched grafts (4.6% vs. 16.1%, p=0.0105); No malignant transformation in both groups
Calabrese et al. [12]	SVF-enriched autologous fat transfer (41)	Non-enriched autologous fat transfer (CG1;64) and reconstruction without fat transfer (CG2;64)	1	NR	84	Annual ultrasound; MRI for suspicious cases	NR	NR	Loco-regional axillary lymph node recurrence: SG 2.4%; CG1 4.7%; CG2 1.6%. No significant risk difference found between groups (p>0.05)	Systemic recurrence: SG 7.3%; CG1 3.1%; CG2 3.1%. SVF did not significantly increase oncological recurrence risk.
Koh et al. [14]	ASC with microfat grafts (5)	Microfat grafts alone (5)	2	29.9 (SG); 12.5 (CG)	15	3D Camera and 3D CT scan	21.71 (SG); 8.32 (CG)	61.1 +/- 13.7 (SG); 34.3 +/- 5.3 (CG); p=0.002	Mean fat uptake was 2.51-fold higher in SG (p=0.046); Mean patient satisfaction/Visual Analogue Scale was higher in SG (4.5 vs 3.1)	NR
Kolle et al. [8]	ASC enriched fat graft (6)	Non-enriched fat graft (6)	2	222.5, 182.5-270 (SG); 260, 240-310.6 (CG); p=0.087	18	MRI	22.8 (SG); 4.7 (CG)	87.6 +/- 17.32 (SG); 44.35 +/- 4.12 (CG)	Median enlargement 2.6 times the initial breast volume in CG; no second procedure needed; better augmentation and cosmetic results in CG	No adverse effects
Peltoniemi et al. [9]	WAL-assisted stem cell-enriched liposuction aspirate (10)	WAL-assisted liposuction aspirate (8)	1	292, 180-438	6	MRI-based volumetric analysis	210.2 (SG); 236.5 (CG)	50.5 +/- 9.586 (SG); 53.55 +/- 8.89 (SG), p= 0.052; statistically not significant	Long-term small oil cysts (one in each group); SG operation was 2–2.5 hours longer	No intra- or post-operative complications; Breast cancer did not occur in either group
Tanikawa et al. [10]	ASC enriched fat graft (7)	Non-enriched fat graft (7)	1	27 (SG) 29 (CG)	6	CT	23.8 (SG); 15.7 (CG)	88 +/- 13 (SG); 54 +/- 20 (CG), p = 0.002	Average increase of surgical time by 45 minutes in SG (p<0.001); Relative risk: SG seven times more likely to produce excellent results, p=0.005	No complications noted
Yin et al. [11]	SVF-assisted fat graft (25)	Non-enriched fat graft (25)	1	55.6 +/-3.8 (SG); 56.5 +/-3.8 (CG), p=0.419	6	3D scanner, Geomagic software and VISIA	42.9 (SG) 31.7 (CG)	77.6 +/-11.6 (SG); 56.2 +/-9.5 (CG), p<0.001	Significantly improved skin texture or VISIA score (15.8+/-7 vs 10.3+/-5, p=0.003) and wrinkles (19.3+/-6.6 vs 10.9 +/-5.5, p<0.001), equal Visual Analogue Scale or patient's cosmetic satisfaction	NR
Glowinski et al. [3]	ASC-enriched fat graft (10, one breast)	Placebo-enriched fat graft (10, one breast)	2	310	12	MRI	167.4 (SG); 173.3 (CG)	54 +/- 11.8 (SG); 55.9 +/- 13.5 (CG), p = 0.566	Fat graft volume retention was not statistically significant; no malignancy was observed in either group	Benign cysts in nine patients were equally distributed between CG and SG
Tissiani et al. [4]	SVF-enriched fat graft (11)	Non-enriched fat graft (8)	1	134.3 (SG); 111.5 (CG)	36 (SG); 16(CG)	MRI-based volumetry	105.8 (SG); 57.3 (CG)	78.8 +/- 74.9 (SG); 51.4 +/- 18.4 (CG), p = 0.31	CD90 expression correlated with survival (p=0.03), cosmetic satisfaction score better in CG group (p = 0.075)	Four cases of fat necrosis in SG (p = 0.103)
Roshdy et al. [13]	SVF-enriched fat graft (15, one side)	Non-enriched fat graft (15, one side)	1	3.3	6	Ultrasound bio- microscopy	3.26 (SG); 0.6 (CG)	70.92 +/-58.09 (SG); 18.93 +/- 19.33, p=0.0001	Loss of augmentation significantly less in SG (p=0.0001); the Visual Analogue Scale or patient satisfaction does not differ significantly (4.3, p=1)	Minimal oedema; no major complications
Li et al. [15]	SVF-enriched fat graft (26)	Non-enriched fat graft (12)	1	17.5	6	CT and Philips Extended Brilliance Workspace	11.5 (SG); 8.1 (CG)	64.8 +/- 10.2 (SG); 46.4 +/- 9.3 (CG), p<0.01	Better cosmetic improvement in SG, p<0.01	No complications noted
Bashir et al. [16]	ASC-enriched fat graft (16)	Non-enriched fat graft (21)	1	NR	6	Ultrasound bio-microscopy	NR	95 +/- 4 (SG); 31 +/- 13 (CG)	Mean patient satisfaction score 4.25 vs 2.52	No fat necrosis observed, one case of cellulitis in the SG
Chang et al. [17]	SVF-enriched fat graft (10)	SVF-enriched fat graft (10)	1	34.4	6	CT and Philips extended brilliance workspace	23.5 (SG); 20.1 (CG)	68.3 +/- 1.7 (SG); 58.5 +/- 1.3 (CG)	NR	No complications noted
Chiu et al. [18]	SVF-enriched fat graft (101)	Non-enriched fat graft (105)	1	310	6	Three-dimensional laser scanning	213 (SG); 210.5 (CG)	68.7 +/- 5.6 (SG); 67.9 +/- 7.9 (CG)	Fat survival rate statistically insignificant	Post-operative complication rate 5.9% vs 3.8%
Yoshimura et al. [2]	ASC-enriched fat graft (3)	Non-enriched fat graft (3)	1	NR	6	Clinical score	NR	NR	Better clinical improvement score in SG, but statistically not significant (p=0.11)	One patient developed fat necrosis in the CG

Graft Survival and Volume Retention

The average volume of fat injected across the cohort was 112.92 ml (95% CI: 40.81-184.98) in the intervention group and 130.57 ml (95% CI: 55.59-205.22) in the control group. Injected volumes ranged from small-volume facial rejuvenation (3.3 mL) to large-scale breast reconstruction (310 mL). Statistical analysis indicated no significant difference in baseline injected volumes between the two cohorts (p = 1.0).

At the final follow-up, the mean residual volume in the enriched graft was 72.40 ml (95% CI 31.4-113.37), compared to 64.29 ml (95% CI 14.32-114.25) in the non-enriched graft. While the intervention group demonstrated a higher mean retention numerically, this difference did not reach statistical significance (p = 0.136). These findings suggest that while there is a trend toward higher retention in the intervention cohort, the current data do not provide sufficient evidence to confirm a statistically significant superior outcome over the control.

**Figure 2:**
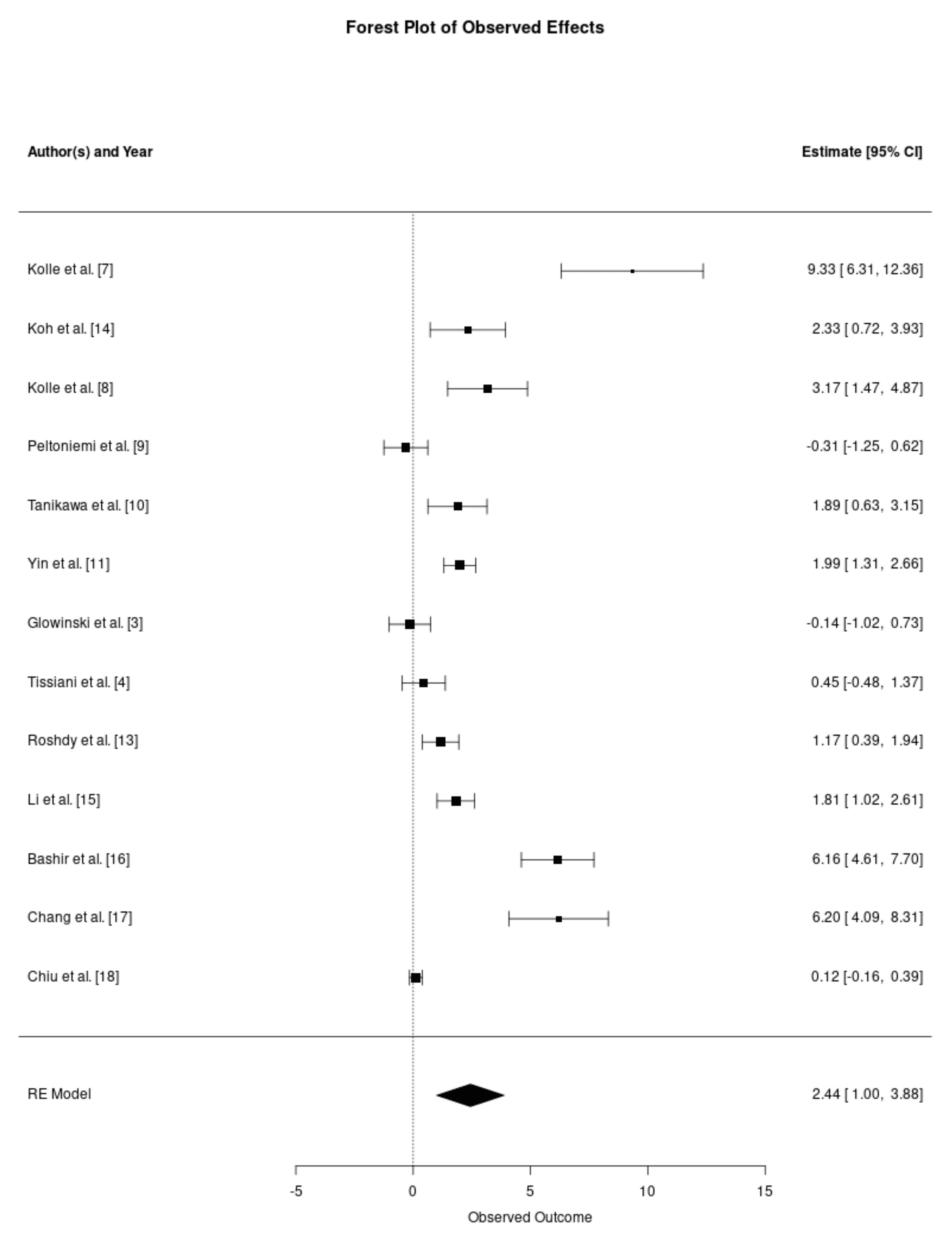
Meta-Analysis of Volumetric Retention Rates Forest plot illustrating the standardised mean difference (SMD) in volumetric retention between cell-assisted lipotransfer and conventional fat grafting. Individual study estimates are represented by squares, with horizontal lines indicating the 95% confidence intervals (CIs). The size of each square corresponds to the relative weight assigned to the study in the random effects (RE) model. The aggregate pooled effect is represented by the diamond at the base, indicating a statistically significant improvement in favour of the enriched group. Statistical outputs for the pooled estimate: number of studies (k = 13); overall effect size (SMD 2.44, 95% CI 1.00–3.88, p = 0.0009); and heterogeneity statistics (tau² = 6.50, I² = 97.19%, H² = 35.60, Cochran's Q = 168.94, p < 0.0001).

Fat survival rate is the primary outcome of the study and was reported in 13 studies. The mean fat survival rate in the CAL-enriched groups was 72.78%, with individual study rates ranging from 50.5% to 95%. Conversely, the non-enriched control groups demonstrated a significantly lower mean survival rate of 45.28%, ranging from 16.3% to 67.9%. The formal meta-analysis indicated a statistically significant improvement in retention favouring the enriched group, with a pooled SMD of 2.44 (95% CI: 1.00-3.88; p = 0.0009). The forest plot of the fat survival rates is provided in Figure 2. Eleven of the 13 analysed datasets demonstrated a positive mean difference in retention rates, ranging from 0.12 to 9.33, with CIs that did not cross the line of no effect, thus confirming statistical significance. Despite this finding, extreme statistical heterogeneity was observed (I² = 97.19%, p < 0.001), suggesting that the magnitude of benefit is context-dependent rather than universal.

Subgroup Analysis by Harvesting Technique

**Figure 3:**
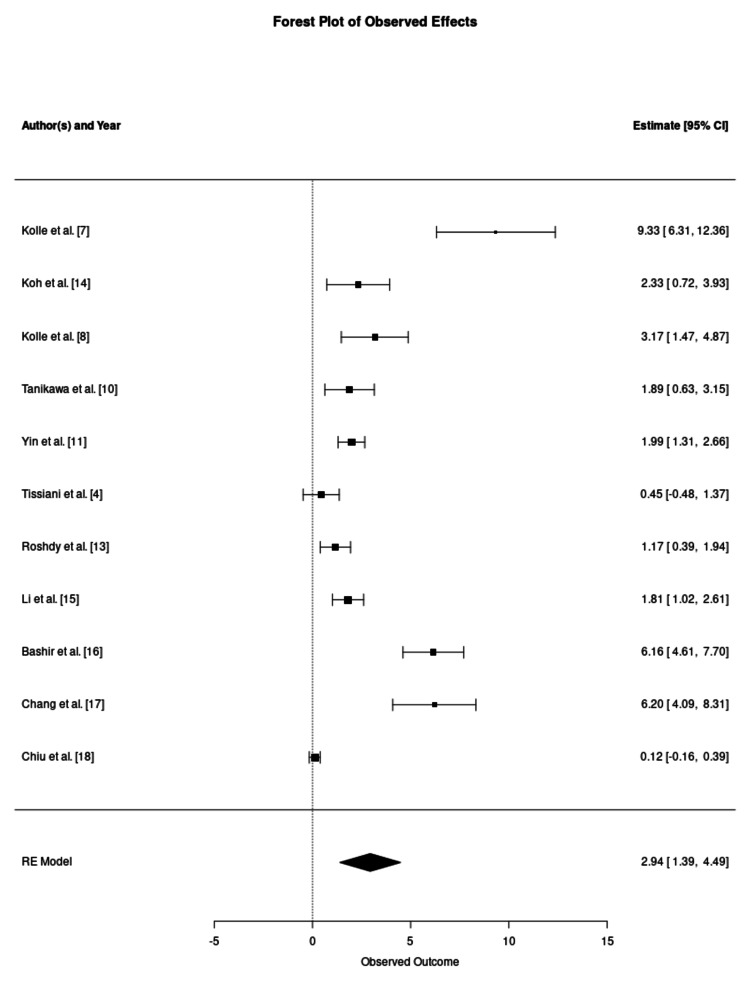
Forest Plot of Graft Retention Using Standard Aspiration Techniques This forest plot synthesises outcomes from 11 studies utilising conventional aspiration methods (e.g., Coleman technique or syringe aspiration). The pooled estimate indicates a statistically significant improvement in graft survival, favouring the enriched groups. Statistical outputs for the pooled estimate: number of studies (k = 11); overall effect size (standardised mean difference: 2.94, 95% confidence interval: 1.39–4.49, p = 0.0002); and heterogeneity statistics (tau^2^ = 6.29, I^2^ = 97.01%, H^2^ = 33.50, Cochran's Q = 158.64, p < 0.0001). RE: random effects

A critical modifier of clinical efficacy identified in this meta-analysis was the choice of adipose harvesting technique. Studies utilising standard aspiration techniques, such as the Coleman method or manual syringe aspiration, demonstrated a pronounced and statistically significant benefit from enrichment, with a pooled SMD of 2.94 (95% CI 1.39-4.49), as demonstrated in Figure 3. For instance, Kolle et al. reported a five-fold higher volume retention in the ASC-enriched grafts (80.9%) compared to the non-enriched control (16.3%) at the four-month follow-up (p < 0.0001) [7]. Koh et al. similarly observed significantly higher retention in the enriched group (61.1% vs 34.3%, p = 0.002) [14]. Kolle et al. demonstrated that this improved retention translated to a 2.60-fold increase in final breast volume compared to a 1.57-fold increase in controls, potentially reducing the need for secondary augmentation surgeries [8]. However, significant statistical heterogeneity was observed (I² = 97.01%), suggesting that while the biological advantage of enrichment is evident in standard aspiration, the magnitude of this effect is highly variable and likely influenced by recipient site characteristics and cell dosing protocols.

**Figure 4:**
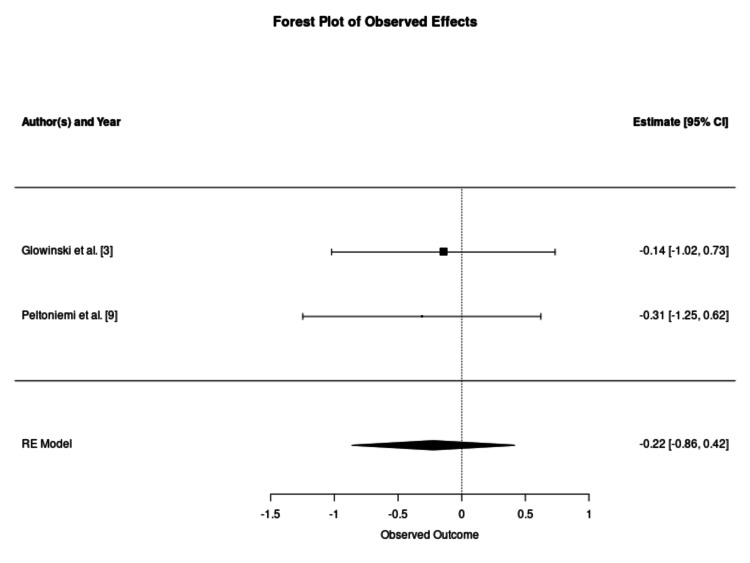
Forest Plot of Graft Retention Using Water-Assisted Lipotransfer This forest plot examines the efficacy of cell enrichment in studies utilising the water-assisted lipotransfer harvesting method. In contrast to standard aspiration, the pooled estimate shows no statistically significant benefit from enrichment (standardised mean difference: -0.22; 95% confidence interval: -0.86–0.42; p = 0.51), as evidenced by the diamond crossing the vertical line of no effect. The heterogeneity for this subgroup was negligible (tau^2^ = 0, I^2^ = 0%, H^2^ = 1, Cochran's Q = 0.0668, p = 0.79), providing consistent evidence that the exogenous addition of regenerative cells does not yield marginal volumetric gains when an atraumatic, closed-system harvesting technique is employed. RE: random effects

On the contrary, studies employing WAL as a primary adipose harvesting technique, such as Peltoniemi et al. and Glowinski et al., found no benefit with stem cell enrichment in fat graft survival (50.5% vs 53.5%, p = 0.052; and 54% vs 56%, p = 0.566, respectively) [3, 9]. Pooled SMD of both of these studies was found to be -0.22 (95% CI -0.86-0.42) and described in Figure 4. These findings support a harvest-quality ceiling hypothesis. It is proposed that the gentle, non-thermal nature of the WAL harvest preserves the viability of native stromal cells and adipocytes so effectively that the exogenous addition of further cells provides no marginal benefit [9]. This analysis suggests that the clinical utility of CAL is context-dependent and may be redundant when high-quality, atraumatic harvesting techniques are employed.

Subgroup Analysis by Recipient Site and Cell Type

**Figure 5:**
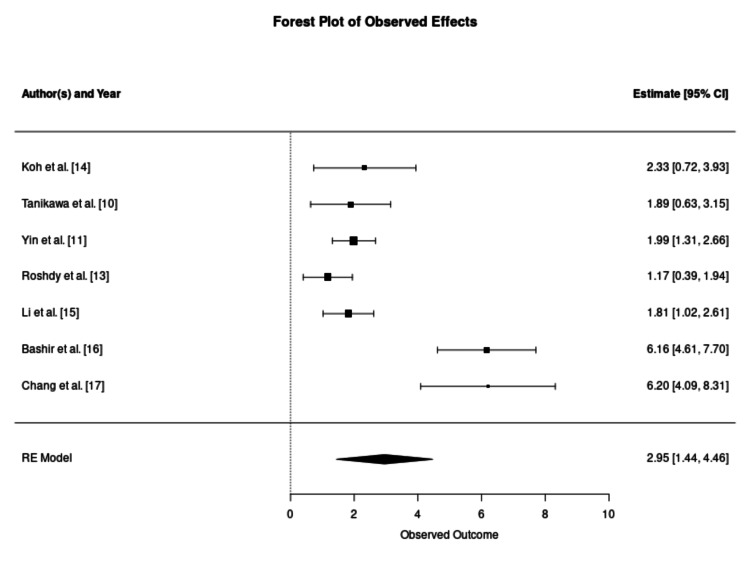
Forest Plot of Graft Retention Subgroup Analysis by Recipient Site (Facial Reconstruction) This forest plot summarises the clinical efficacy of cell enrichment in facial applications, including cosmetic rejuvenation and reconstructive procedures for hemifacial atrophy. The analysis reveals a robust and statistically significant benefit for enriched grafts (standardised mean difference: 2.95; 95% confidence interval: 1.44–4.46; p = 0.0045). The heterogeneity (tau^2 ^= 3.7, H^2^ = 14.2,  I^2^ = 92.96%, Cochran's Q = 47.22, p <0.0001) is lower than the overall pool, indicating that the facial recipient bed provides a more consistent environment for cell-assisted survival than larger-volume sites. RE: random effects

**Figure 6:**
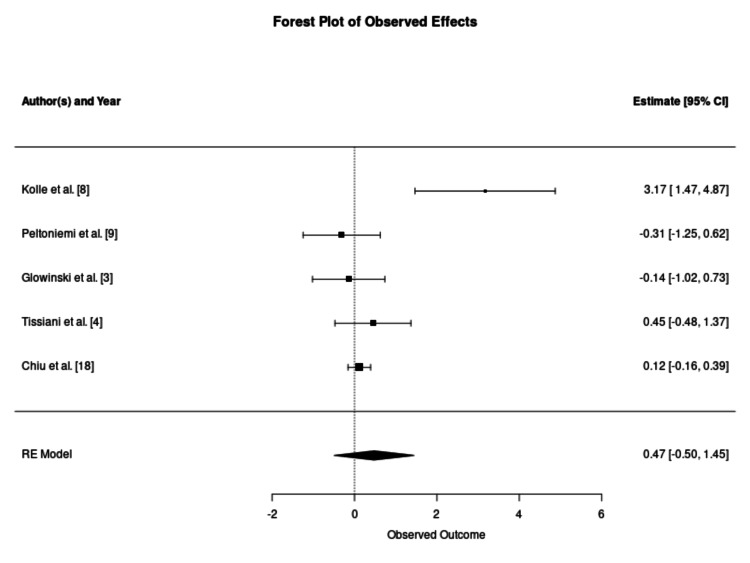
Forest Plot of Graft Retention Subgroup Analysis by Recipient Site (Breast Reconstruction) This forest plot examines the impact of cell enrichment in large-volume breast reconstruction. In contrast to facial sites, the aggregate data show no statistically significant survival advantage for enriched grafts in the breast (standardised mean difference: 0.47; 95% confidence interval: -0.50–1.45; p = 0.23). Moderate heterogeneity was observed (tau^2^ = 0.98, H^2^ = 7.38, I^2^ = 86.46%, Cochran's Q = 13.95, p=0.0074), suggesting that in large-volume transfers, physiological factors such as the graft-to-recipient surface area and oxygen diffusion limits may override the potential paracrine benefits of cell enrichment. RE: random effects

Enrichment effects varied significantly by anatomical region. Facial applications (including hemifacial atrophy and cosmetic rejuvenation) showed robust survival gains with a pooled SMD of 2.95 (95% CI: 1.44-4.46; p = 0.0045), as described in Figure 5. Conversely, large-volume breast reconstructions showed no statistically significant aggregate benefit from enrichment (SMD 0.47; 95% CI: -0.50-1.45; p = 0.23), as represented in Figure 6. This finding suggests that in large-volume breast grafting, the graft-to-recipient surface area ratio is the primary limiting factor. Even with stem cell enrichment, the central portion of large fat grafts likely remains vulnerable to necrosis due to the physical limits of oxygen diffusion before neovascularisation can be established. On the contrary, the compact nature of facial compartments and the high baseline vascularity of the face provide a more receptive environment for stem cell-mediated angiogenesis.

**Figure 7:**
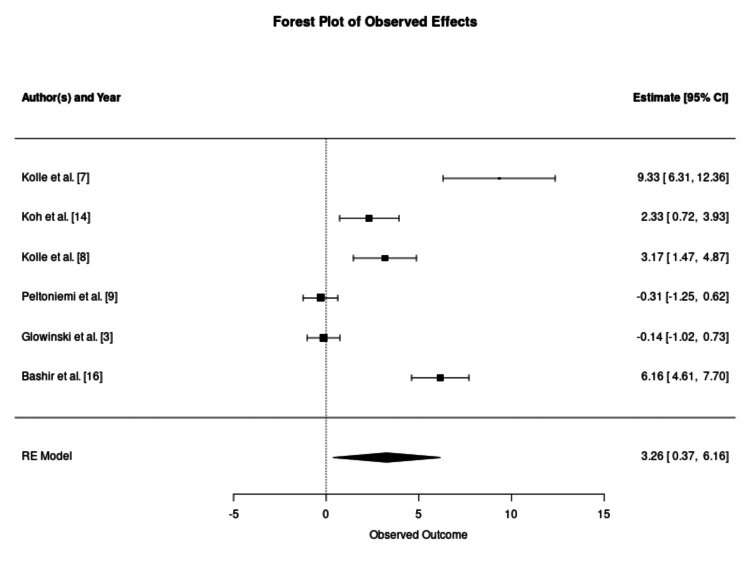
Forest Plot of Graft Retention Using Adipose-Derived Stem Cells as an Enrichment Modality This forest plot evaluates the efficacy of grafts enriched specifically with culture-expanded adipose-derived stem cells. The meta-analysis demonstrates a statistically significant survival advantage for the enriched cohort (standardised mean difference: 3.26; 95% confidence interval: 0.37–6.16; p = 0.048). The observed heterogeneity (I^2^ = 94.6%) suggests that even with standardised cell expansion, clinical outcomes are significantly influenced by individual study protocols and recipient site conditions. RE: random effects

**Figure 8:**
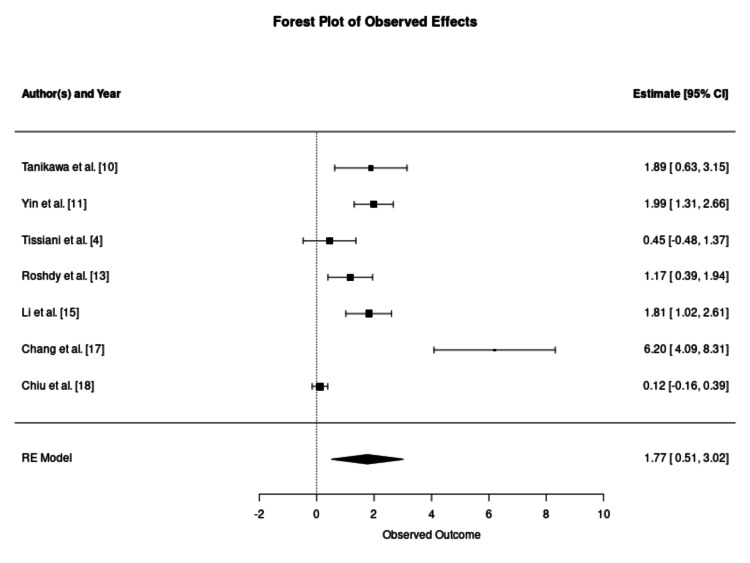
Forest Plot of Graft Retention Using Stromal Vascular Fraction as an Enrichment Modality This forest plot illustrates the outcomes of grafts enriched with stromal vascular fraction at the point of care. Enrichment with stromal vascular fraction yielded a statistically significant improvement in volume retention compared to controls (standardised mean difference: 1.77; 95% confidence interval: 0.51–3.02; p = 0.01). Moderate-to-high heterogeneity (I^2^ = 91.4%) was noted, likely reflecting the inherent biological variability of non-expanded, heterogeneous cell populations across different patient donors. RE: random effects

Regarding cell type, both subgroups favoured enrichment, though effect magnitudes differed. The pooled survival rate for cultivated ASCs showed a significant advantage over controls (SMD: 3.26; 95% CI: 0.37-6.16; p = 0.048), as suggested in Figure 7. The SVF subgroup also favoured enrichment but displayed a wider variance in outcomes (SMD: 1.77; 95% CI: 0.51-3.02; p = 0.01), as described in Figure 8.

*Histological Analysis* 

Compared with the control group, Kolle et al. demonstrated higher amounts of adipose tissue and newly formed connective tissue, suggesting fibroblast-like appearance and differentiation capabilities of ASC [7]. Notably, regarding vasculogenesis, they found no statistically significant increase in vessel density between the enriched and control groups (20.86 vs 26.32 vessels/mm², p = 0.39) [7]. While these results are based on a limited dataset, they suggest that the biological benefit of ASC enrichment may be more closely associated with paracrine-mediated tissue preservation and stromal remodelling than with a purely numerical increase in macroscopic vasculogenesis.

*Skin Quality and* *Aesthetic Outcomes*

The impact of cell enrichment on skin rejuvenation and cosmetic quality was evaluated using both objective and subjective measures. Yin et al. recorded facial skin quality pre-and post graft by using a video image skin interpretation and analysis (VISIA) skin detector [11]. The study found superior results for the structural parameters in the enriched group. Specifically, wrinkle and texture scores were significantly higher in the SVF-enriched group than in the control group: 19.3 +/- 6.6 versus 10.9 +/- 5.5 (p < 0.001) and 15.8 +/- 7.0 versus 10.3 +/- 5.0 (p = 0.003), respectively [11].

In contrast to these findings, two studies reported that cosmetic benefits were significantly superior in the non-enriched control groups [4, 8]. This discrepancy highlights the complexity of assessing quality versus quantity, as superior volumetric retention does not always translate to a better aesthetic or skin-surface result across all patient populations and anatomical sites.

Patient and Professional Satisfaction

Patient satisfaction was assessed using a five-point Visual Analogue Scale. While satisfaction was generally high across both cohorts (p = 0.52), individual study findings were heterogeneous. Koh et al. reported significantly higher mean satisfaction in the enriched group compared to the control (4.5 vs. 3.1) [14]. Conversely, Yin et al. and Tissiani et al. found that the majority of participants in both groups were equally satisfied with the aesthetic outcomes [4, 11]. Interestingly, Tissiani et al. noted a non-significant trend (p = 0.075) where control group patients reported slightly higher satisfaction scores (8-10) than those in the stem group (5-10) [4]. Despite this, most patients across both arms indicated they would undergo the procedure again (p > 0.999) and would recommend it to others (p = 0.546) [4].

Professional satisfaction, assessed by 10 independent board-certified surgeons in a blinded clinical photo review by Kolle et al., revealed significantly superior clinical results in the CAL-treated group compared to controls, providing objective validation of the enrichment technique’s aesthetic advantage [8].

Complication Rates

Complications were categorised as minor (erythema, mild oedema, hematoma, local pain at incision site, and oil cyst) and major complications (infection, tissue loss, skin necrosis, fibrosis, severe oedema, pain spreading beyond injection site, cellulitis, fat embolus, and embolus causing blindness). Overall, six studies reported no adverse events in either cohort.

Fat necrosis was identified as the most significant concern in autologous fat grafting and was described in four studies. The overall safety profile remained in favour of enriched grafting. Fat necrosis was notably lower in CAL groups (e.g., Kolle et al., 4·6% vs 16·1%, p = 0·0105) [7]. On the contrary, Tissiani et al. reported fat necrosis among four patients in the stem group, while it was not noted in any patients in the control (𝑝 = 0.103) [4]. However, this difference was not statistically significant (p = 0.92), indicating that while enrichment may offer benefits in specific contexts, it does not universally eliminate the risk of necrosis.

Other reported complications were minor. Oil cysts were observed at equal rates between the two groups [3,9]. These cysts are known to develop one to two years after lipotransfer and can cause anxiety, but are easily treated by ultrasound-assisted aspiration, if needed.

Oncological Safety and Malignancy Potential

Given the angiogenic potential of progenitor cells, the risk of tumour induction or recurrence, particularly in breast reconstruction, remains a subject of debate [9]. However, available clinical evidence does not demonstrate an increased oncological risk within reported follow-up periods. A pooled analysis of four studies indicated that enrichment did not significantly increase the risk of loco-regional recurrence (p = 0.42) [4, 7, 9, 12]. 

Most notably, the regression analysis performed by Calabrese et al. indicated that the use of concentrated SVF was not a significant independent risk factor for either local recurrence or systemic metastasis [12]. In their cohort, the non-enriched control group actually exhibited a higher raw number of systemic recurrences, including bone and pulmonary metastases, compared to the SVF-enriched group.

Nevertheless, the follow-up period represents a significant limitation in the oncological context. The mean follow-up duration across the included studies was 16.2 months. While this window is sufficient for assessing volumetric graft retention, it is arguably insufficient for a definitive oncological safety profile. Breast cancer recurrences often manifest in a five-to-10-year window; therefore, the current evidence can only confirm safety within the short-to-medium term. Future research must prioritise long-term surveillance data to determine if these regenerative therapies remain safe over the full decade following reconstruction.

Operative Efficiency and Economic Analysis

The biological advantages of enrichment are offset by increased resource utilisation. Yin et al. provided a detailed economic breakdown, revealing that while basic hospital stay costs were equivalent between cohorts, the total procedure cost was significantly higher in the CAL-enriched group ($7,390) compared to the control group ($6,601) (p < 0.05). This cost disparity was largely driven by the utility of expensive enzymatic processing kits and the extended use of operating room resources [11]. 

Anaesthesia time in the CAL-enriched and control groups were approximately four and 2.5 hours, respectively [11]. Peltoniemi et al. observed a similar trend with prolonged operation time by 2-2.5 hours and higher costs in the stem cell-enriched group [9]. Tanikawa et al. reported that for the control group, the mean surgical time was 80 minutes, while for the experimental group, there was an average increase of 45 minutes (p < 0.001) [10].

Risk of Bias in Individual Studies

The methodological quality of the included studies was independently assessed by two reviewers using the EPHPP tool [19]. This instrument was selected for its versatility in evaluating diverse study designs, including both RCTs and cohorts. Each study was evaluated across six domains: selection bias, study design, confounders, blinding, data collection methods, and withdrawals or dropouts. Based on these parameters, a global rating was assigned. Studies with no weak ratings were classified as strong, those with one weak rating as moderate, and those with two or more weak ratings as weak.

The detailed assessment is presented in Table 4. Three studies [3, 7, 8] achieved a strong global rating, four were rated as moderate [11, 13, 14, 16], and the remaining eight studies received a weak global rating [2, 4, 9, 10, 12, 15, 17, 18]. Notably, 62% of the studies received a strong rating for study design, and 75% were rated strong for data collection. This reflects a significant improvement in the field, as researchers have shifted away from subjective patient satisfaction surveys toward objective, measurable analysis using MRI and 3D-CT scans.

**Table 4:** Methodological Quality and Global Ratings of Included Studies Methodological quality assessment using the Effective Public Health Practice Project (EPHPP) tool [19]. Studies are rated as Strong, Moderate, or Weak in each domain. A global rating of Strong is assigned when there are no weak ratings; Moderate is assigned if there is one weak rating, and those with two or more weak ratings are considered weak.

Study	Selection bias	Study design	Confounders	Blinding	Data collection	Withdrawal	Global rating
Kolle et al. [7]	Strong	Strong	Strong	Strong	Strong	Strong	Strong
Calabrese et al. [12]	Moderate	Moderate	Moderate	Weak	Strong	Moderate	Weak
Koh et al. [14]	Strong	Strong	Strong	Moderate	Strong	Strong	Moderate
Kolle et al. [8]	Strong	Strong	Strong	Moderate	Strong	Strong	Strong
Peltoniemi et al. [9]	Moderate	Moderate	Weak	Weak	Strong	Moderate	Weak
Tanikawa et al. [10]	Moderate	Strong	Strong	Weak	Moderate	Moderate	Weak
Yin et al. [11]	Moderate	Strong	Strong	Moderate	Strong	Strong	Moderate
Glowinski et al. [3]	Strong	Strong	Strong	Strong	Strong	Strong	Strong
Tissiani et al. [4]	Moderate	Moderate	Moderate	Weak	Moderate	Strong	Weak
Roshdy et al. [13]	Moderate	Strong	Strong	Weak	Strong	Strong	Moderate
Li et al. [15]	Weak	Moderate	Moderate	Weak	Strong	Strong	Weak
Bashir et al. [16]	Moderate	Strong	Strong	Weak	Strong	Strong	Moderate
Chang et al. [17]	Weak	Moderate	Moderate	Weak	Strong	Strong	Weak
Chiu et al. [18]	Weak	Moderate	Moderate	Weak	Strong	Strong	Weak
Yoshimura et al. [2]	Weak	Moderate	Weak	Weak	Moderate	Moderate	Weak

However, biases remain. Blinding was the most significant weakness, with 62% of studies rated as weak in this domain. This is largely attributable to the logistical impossibility of blinding the operating surgeon to the enrichment process. Furthermore, selection bias was rated as moderate in 56% of studies, a reflection of the single-centre nature of most trials and strict inclusion criteria. Despite these specific deficits, the consistent finding of superior graft retention across the two out of three strongly rated RCTs suggests that the efficacy of cell-assisted lipotransfer is a biological reality rather than an artifact of methodological bias.

Discussion

The meta-analysis results demonstrate that while CAL is associated with an overall increase in graft survival (72.78% over 45.28%), its clinical utility is characterised by significant variability. The following synthesis evaluates these outcomes through a critical, analytic lens, prioritising clinical context over pooled statistical metrics.

Instability of Effect Size Estimates

The extreme statistical heterogeneity identified in this study (I^2^ = 97.19%) underscores that effect size estimates for CAL remain inherently unstable. The calculated SMD of 2.44 is exceptionally high, an artifact of measurement inconsistency across studies rather than a uniform biological effect. Because studies utilised different assessment modalities across variable timelines, the magnitude of the SMD must be interpreted with caution. Our subgroup analyses further clarify this by showing that the benefit is highly dependent on the anatomical site and harvest technique. Therefore, until a therapeutic window for ASC concentration is defined, meta-analysis will always struggle with such variance. Furthermore, while the aggregate diamond favours enrichment, the wide range of reported retention from 50.5% to 95% suggests that a mean survival rate is a poor predictor for individual surgical success. Consequently, the pooled magnitude should be viewed as a theoretical indicator of biological potential rather than a guaranteed clinical outcome.

Clinical Context as a Primary Determinant

A nuanced finding of this review is that clinical context matters more than pooled magnitude when determining the efficacy of enrichment. Subgroup analysis reveals that anatomical site and recipient bed vascularity are more definitive predictors of graft take than the mere presence of exogenous cells. In facial recontouring, enrichment provided a robust survival advantage for small-volume injections (<100ml) in highly vascularised facial tissue. On the contrary, the benefit of large-volume breast grafting was statistically negligible. In these scenarios, the physiological limits of oxygen diffusion and the graft-to-recipient surface area ratio likely override the paracrine influence of added stem cells.

Absence of Uniform Superiority

Based on the evidence synthesised, CAL cannot be considered uniformly superior to conventional autologous fat grafting. The harvest-quality ceiling observed in the WAL subgroup demonstrates that high-quality, atraumatic harvesting may preserve the native regenerative niche so effectively that further cell supplementation becomes redundant. Furthermore, the clinical translation of CAL is currently hindered by a lack of robust economic and histological data. Our analysis of operative efficiency and cost was limited to a few specific trials, and while these data suggest significantly higher resource utilisation, further large-scale, multicenter studies are required to confirm the cost-effectiveness and microstructural impact of cell enrichment.

Furthermore, it must be acknowledged that a significant proportion of the studies included in this meta-analysis received a weak global rating for methodological quality. This is primarily due to the inherent challenges of blinding in surgical trials and the prevalence of small, single-centre cohorts. Consequently, while the statistical trend favours CAL, these findings should be viewed as suggestive of a biological advantage rather than a definitive endorsement of clinical superiority. The strength of our conclusions is necessarily tempered by the quality of the current evidence base, highlighting the urgent need for more robust, high-power RCTs. 

The Paracrine Paradigm

Historically, the efficacy of CAL was attributed to de novo vasculogenesis. However, the high-quality histological data from Kolle et al. challenge this paradigm [8]. Despite superior volume retention, enriched grafts did not demonstrate a higher vessel density than controls. This finding suggests a shift towards the paracrine hypothesis. Rather than structurally differentiating into new vasculature, transplanted ASCs may function as cellular modulators. By secreting potent anti-apoptotic and pro-angiogenic factors during the immediate post-transplant hypoxia, they likely preserve the viability of mature adipocytes until the host vascular network can act. This biostimulatory property is particularly valuable in the treatment of radiation-induced fibrosis and scarred recipient beds (e.g., Parry-Romberg syndrome), where the graft serves as both a filler and a biological modulator.

Limitations of the Study

This review is limited by the heterogeneity of the included studies. A major shortcoming in virtually all analysed papers is the near lack of properly described standardised procedures. Critical methodological parameters, such as exact enrichment ratios, CD marker expression, and specific centrifugation parameters, were frequently not reported. This pervasive lack of detail, combined with the absence of a standardised therapeutic dose for ASCs, severely restricts the comparability between studies and contributes directly to the high statistical heterogeneity observed.

Volume retention is the goal of fat grafting, but it is a highly challenging parameter to measure. While several studies used validated imaging methods (CT, MRI, 3D surface imaging), the lack of a standardised therapeutic dose for ASCs and diverse measurement protocols complicate direct comparison. The fat injection volume was often based on the patient’s needs and varied from patient to patient, thereby potentially introducing bias. Furthermore, it is critical to note that none of the papers disclosed unbiased reliability metrics, such as inter- and intra-observer variation. This omission reduces the objectivity of the reported outcomes, as measurements without reliability coefficients are prone to subjective bias.

The sample sizes of some eligible studies were also relatively small, and therefore, may not have been enough to confirm their outcomes. Alongside this, the limited follow-up period may have resulted in underestimated complication rates. This prevented the performance of a statistically robust meta-regression. Consequently, categorical subgroup analyses were utilised as a more reliable method to explore the sources of heterogeneity within the current data limits. The inclusion of observational study designs alongside RCTs further contributed to the observed statistical heterogeneity. Finally, the predominance of breast and facial studies limits the generalisability of these findings to other body regions. 

## Conclusions

CAL offers a clear biological advantage in specific scenarios, notably in hostile recipient beds or when standard aspiration techniques are used. However, it is not a one-size-fits-all solution. The choice to utilise CAL must be a tailored clinical decision that weighs the specific anatomical site and harvesting technology against the increased operative time and cost. Future research should prioritise the standardisation of cell dosing to establish a predictable, dose-dependent response curve for clinical use.
